# Drug treatment service procurement: A systematic review of models, goals, and outcomes

**DOI:** 10.1177/14550725231157503

**Published:** 2023-06-22

**Authors:** Taina Schneider, Kristiina Kuussaari, Petri Virtanen

**Affiliations:** 8130 University of Vaasa, 3837 Finnish Institute for Health and Welfare, Helsinki, Finland; 3837 Finnish Institute for Health and Welfare, Helsinki, Finland; 8130 University of Vaasa, Itla Children's Foundation, Vaasa, Finland

**Keywords:** co-creation, complexity, drug treatment services, public procurement, public value, systematic literature review

## Abstract

**Aim:** To explore the goals and outcomes of public procurement of drug treatment services in OECD countries. The study explores how these complex services are procured and delivered. **Methods and data:** A systematic review of the literature (1990–2020) identified four partly overlapping models of drug treatment service procurement that are here labelled traditional, value-based, outcome-based, and innovative. **Results:** Even though different forms of drug treatment services procurement are common, only 12 empirical studies that focused on procurement were found. The four models differ in their approaches to design and performance specifications and the role of competition and collaboration in the co-creation of value. **Conclusions:** Competition and incentives improve neither the efficiency nor the quality or the outcomes of complex drug treatment services. Whereas many studies focus on payment mechanisms, there are important research gaps that relate to the co-creation of value with and for the service-users and other stakeholders.

## Introduction

Public procurement affects everyone's lives. “Economic, social and environmental benefits of public programmes and projects to a country and its citizens depend very much on how public procurement is managed, conducted and controlled” (Khan, 2018). Drug treatment services (DTS) provided in OECD countries are often delivered through public funding and therefore those services are subject to public procurement regulations. However, little is known about how these complex DTS are procured or what the outcomes of that procurement are (e.g., Babor et al., 2019; Ritter & Van De Ven, 2019). This systematic review explores procurement models and mechanisms relating to DTS and underlines the outcomes and usefulness for individuals and society. This knowledge is essential to organise DTS in a way to create value for service-users and society. In this study, procurement is viewed from a broad perspective as a cycle of functions included in the broader strategic commissioning and planning of publicly funded services, and that encompasses a purchasing cycle (Murray, 2009). The main phases of the procurement cycle are planning and preparation, tendering, and contracting (EU, 2019; OECD, 2009).

The topic of procurement of public services has attracted significant attention in recent decades. Current procurement models have resulted from global trends and dominant governance models related to public administration and their country-specific implementation as a part of health and social reforms. Since the late 1980s, new public management (NPM) has strongly influenced procurement practices because of its focus on marketisation and competition, management of organisations’ internal performance, outputs, and effectiveness (Barraket et al., 2016; Osborne, 2021). However, when facing closed organisational systems, the NPM and traditional forms of procurement fail to address complex issues such as wicked societal problems. The main characteristics of these problems are challenge of problem definition, non-resolvability, and multi-actor environment, which require a holistic approach and a collaborative strategy (Crowley & Head, 2017; Head & Alford, 2015; Klijn, 2008; Raisio et al., 2019; Rittel & Webber, 1973).

Under New Public Governance (NPG), the public service delivery focus is on external networks, partnership, co-production, and co-creation with service-users and other stakeholders. The other key focus is on outcomes, that is, value for service-users and other stakeholders. One fundamental point of departure in this process has been to treat public interventions as value-creating services, not products (Osborne, 2010, 2021; Sorrentino et al., 2018; Strokosch & Osborne, 2020; Tuurnas et al., 2019). The emphases of NPG have also strongly reflected public service procurement, particularly social, outcome-based, and innovative forms of procurement (Barraket et al., 2016; Tirronen, 2020; Torvinen & Ulkuniemi, 2015).

Parallel to the above evolution of public service delivery and procurement, traditional accountability has more or less shifted from the vertical to the horizontal form. In other words, alongside the traditional (legal and political) accountability, are also to be found the systematic performance measurement from NPM and multiple forms of accountability from the collaborative NPG. In addition, the roles of service-users are changing; NPM focuses on consumers/customers and the NPG on co-creators of services (e.g., Agger & Lund, 2017; Schillemans, 2011; Torfing & Triantafillou, 2013; Virtanen et al., 2018).

Drug treatment services are part of the health and social sector, and both the services provided and their procurement illustrate forementioned complex societal issues (Klijn, 2008; Raisio et al., 2019; Unlu et al., 2021). Problematic drug use often has severe consequences, such as high morbidity and mortality rates (Alho et al., 2020; EMCDDA, 2021; Gomes et al., 2018). Morbidity is frequently related to infectious diseases and mental health problems and somatic disorders alike (EMCDDA, 2021; Kadri et al., 2019; Whiteford et al., 2013). Illicit opioid use and non-medical use of prescription opioids have caused increased mortality rates in the United States and Australia as well as in Europe (Alho et al., 2020; Gomes et al., 2018). Homelessness, lack of education, unemployment, criminality, and other social problems among service-users are also commonplace (de Espíndola et al., 2020; Kuussaari & Hirschovits-Gerz, 2015).

**Figure 1.**
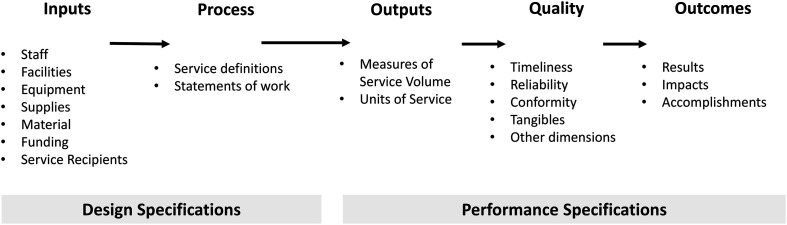
The expanded systems framework (Martin, 2005).

In this study, the expanded systems framework (Martin, 2005) was deployed to identify the different emphases of DTS procurement (see Figure 1). If the main phases of procurement predominantly emphasised design specifications (inputs and process) and outputs, we interpreted procurement to be of the traditional form, and the orientation to be derived from the NPM. If the emphasis was mostly on outcomes, we interpreted that procurement is conducted within the framework of the NPG and the focus is on the co-creation of value with and for service-users and other stakeholders.

The aim of the present study was to identify knowledge gaps in DTS procurement processes and thus to provide practical guidance on how to organise and maintain complex procurement processes in public services in a way that creates value for service-users. The study is structured based on the following questions:

1. How are DTS procured?
2. What are the goals of DTS procurement, and how is goal attainment assessed?
3. What are the outcomes of DTS procurement?

The study progresses as follows: first, we describe the method and data employed; we then present four models of DTS procurement; and finally, we discuss the lessons learned and conclude with some pointers for future research and practice.

## Methods and data

This systematic review was conducted using the Preferred Reporting Items for Systematic Reviews and Meta-Analyses (PRISMA) statement (Liberati et al., 2009; Moher et al., 2015; Rojon et al., 2021). The Population, Intervention, Comparator, Outcome (PICO) framework defined the research questions and search terms. In this study, the key elements of PICO were people who use drugs, public procurement of social and health services, different procurement practices, and outcomes of procurement (Xiao & Watson, 2019).

**Figure 2.**
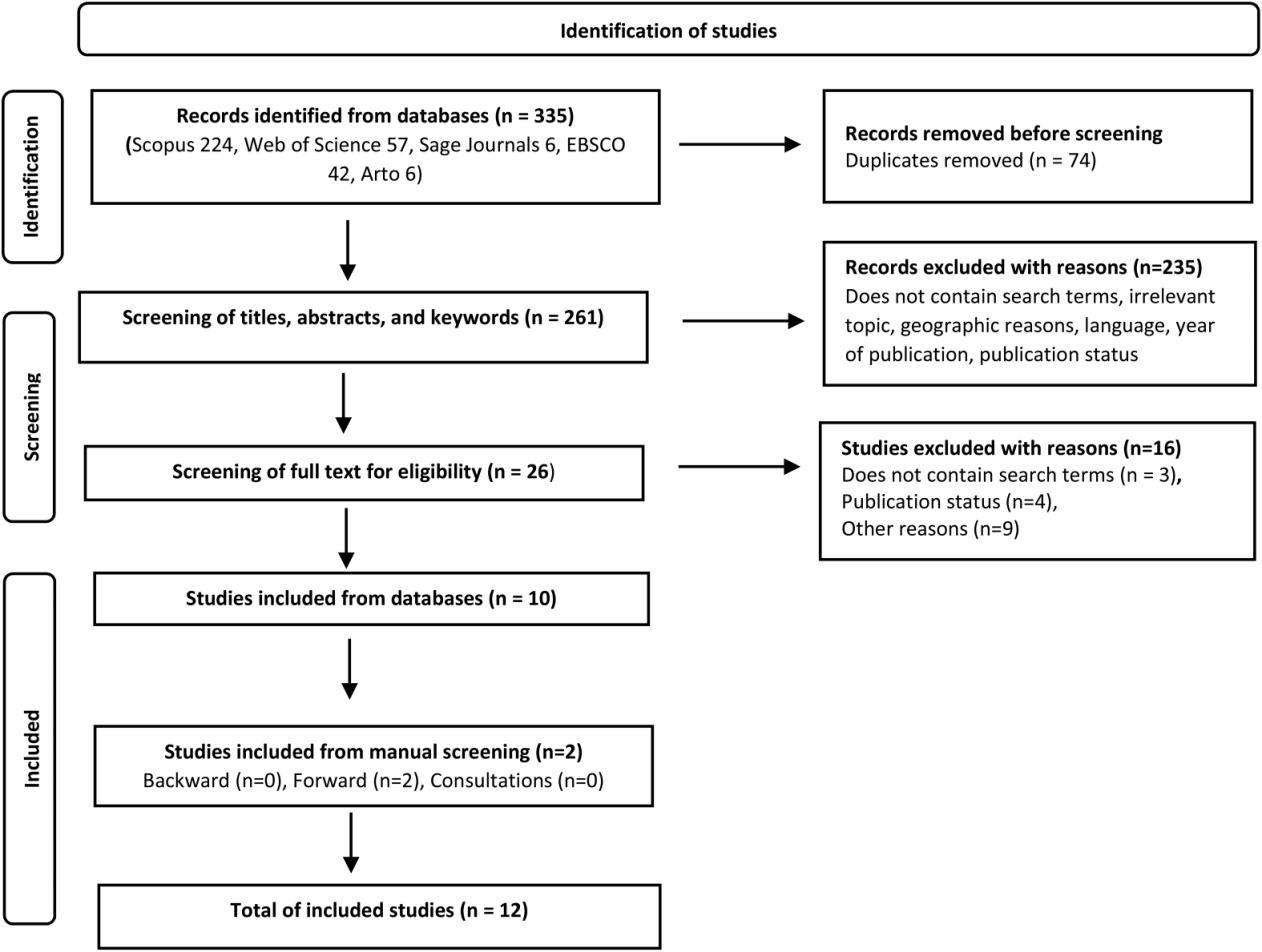
The search strategy of the systematic review based on the PRISMA statement flow diagram.

In spring 2020, we examined previous research, did a test search, and consulted an information specialist to formulate search terms that offered a good balance between breadth and depth (Fisch & Block, 2018). We also paid attention to cultural differences between countries and used synonyms, abbreviations, and related terms (Xiao & Watson, 2019). Based on the above, the Boolean search terms used were (“drug addict” OR “drug abuser” OR “substance abuser” OR “drug misuser” OR addict) AND (“public procurement” OR “competitive procurement” OR procurement OR purchasing OR outsourcing OR privatisation) AND (treatment OR service). The first eligibility criterion was that a study should contain the required search terms in the title, abstract, or keywords. The second criterion was that the study should deal with the procurement of DTS as part of commissioning publicly funded services in OECD countries. The review includes empirical studies published after 1990 in peer-reviewed scientific journals written in English or Finnish. Grey literature (e.g., reports) was excluded to ensure transparent and systematic selection and the quality of the included studies (Kraus et al., 2020). To identify articles, we used five electronic databases: Scopus, Web of Science, Sage Journals Online, EBSCO, and Arto. The final search was completed in May 2020. The search strategy is presented in Figure 2.

A total of 335 studies were identified in the database search. Duplicates were removed, and the authors screened 261 studies based on title, abstract, and keywords, after which 235 articles had been excluded (see Figure 2). In the second stage of screening, the remaining 26 studies were assessed more carefully. Ten studies met all the inclusion criteria and were included in this systematic review. In April 2021, backward and forward searches of the included articles were completed. The first author also requested the advice of three well-known authors in the field. Finally, a total of 12 articles were included in our systematic review. After the identification process, we assessed the quality of included studies. We used the quality checklist, which was developed based on existing guidelines and checklists for qualitative and quantitative studies (Rojon et al., 2021; Spencer et al., 2003; Tranfield et al., 2003; Tufanaru et al., 2020). The first author read and assessed all 12 studies independently. The second and third author each read and assessed six studies. The initial assessments of the author team largely conformed, and minor differences were discussed and resolved together.

**Figure 3.**
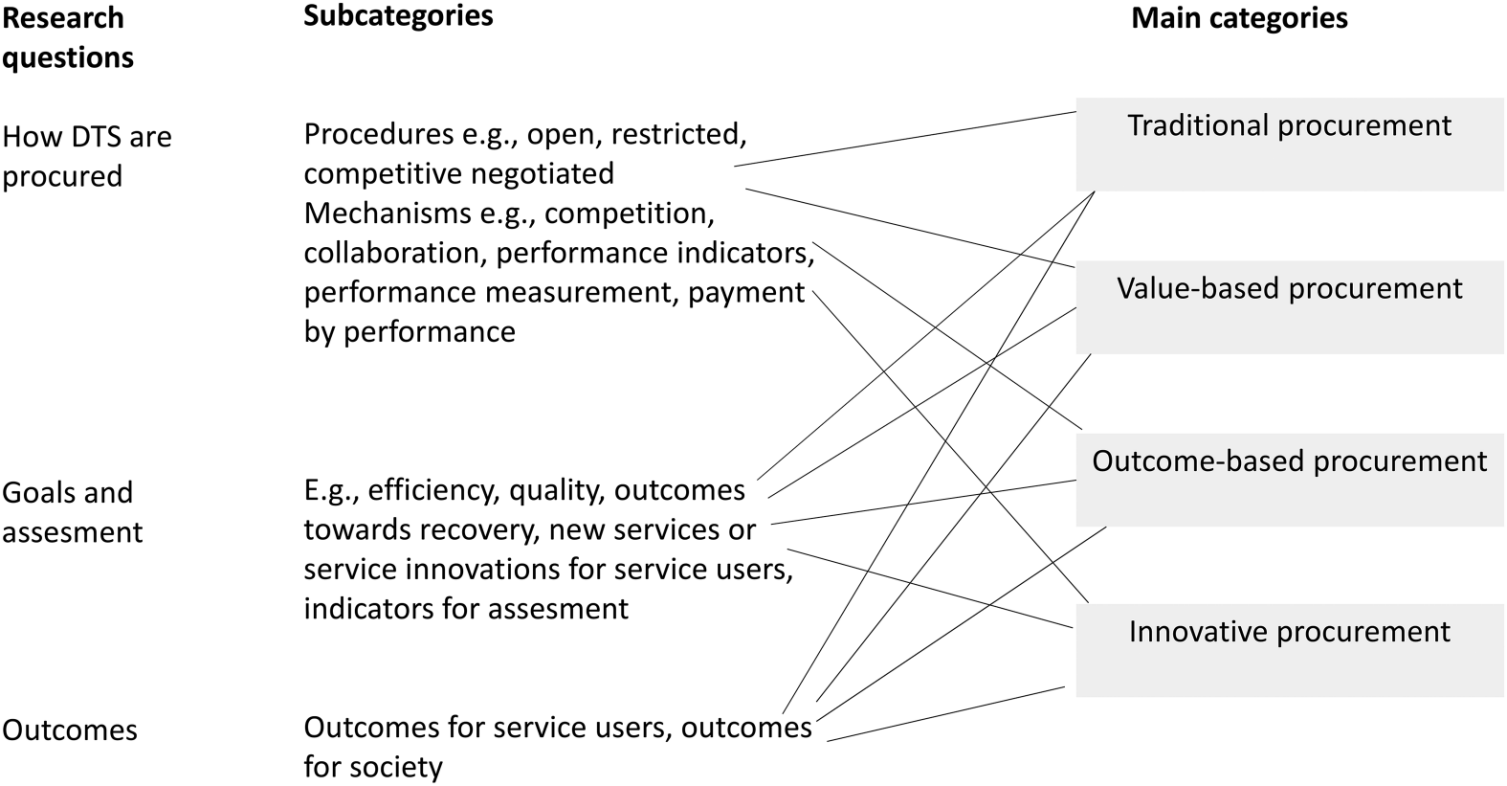
The content analysis process.

We used content analysis (Mikkonen & Kääriäinen, 2019; Rojon et al., 2021, 206) guided by the research questions and our theoretical framework. The first author reread all of the included studies and coded the text that was linked to the research questions. The coded text was then exported to the codebook. The text was next arranged into sub-categories based on the words that described the procurement mechanisms, goals, and outcomes. Based on the sub-categories and guided by our theoretical framework, we identified four main categories of DTS procurement (Figure 3). During the analysis, the research team discussed emergent issues and made decisions collectively.

Table 1 presents some basic information on the studies included in this systematic review. Most of the studies (n = 8) were published in journals addressing addiction (*n*=5). The majority of studies did not specify the phase (planning, tendering, or contracting) of the procurement cycle or the procurement procedure, which were topics we were interested in. Even so, in many studies, procurement was described as if it were a tool of NPM ideology.

**Table 1.** Overview of studies in the systematic review.

Focus	Concepts	Methods and data	Country and context	Study
Practitioners’ experiences of payment by results	Outcome-based commissioning, PbR	Qualitative; single case (focus group in 2016, 11 participants)	England, alcohol and drug treatment sector, one therapeutic community	Gosling (2018)
The effects of payment by results on recovery outcomes	P4P, PbR, outcomes	Quantitative (NDTMS data, Police national computer records and ONS mortality records from 2010 to 2014, service-users self-reporting)	England, areas participating (8) or non-participating (143) to P4P, publicly funded drug services	Jones et al. (2018)
Procurement of innovation in social services	Procurement, service innovation	Qualitative; single case (documents related to procurement process from 2011 to 2012, 21 interviews)	Finland, the city of Helsinki, social services for substance abusers	Pelkonen & Valovirta (2015)
Effectiveness of value-based purchasing for treatment engagement and retention	Value-based purchasing, incentives, performance-based contracting	Quantitative (administrative data from FY2005–FY2011)	United States, Maine, substance use treatment for outpatient and intensive outpatient	Reif et al. (2021)
Purchasing levers to increase the use of evidence-based practices	Purchasing, purchasing levers	Quantitative and qualitative (survey, 51 key informant interviews in 2008)	The United States, 51 states, publicly funded substance abuse treatment	Rieckmann et al. (2011)
Conceptual schema for purchasing arrangements	Treatment purchasing, fee for service, competitive tendering	Qualitative (literature review in 2013 and 2014, group interviews in 2013, 190 participants, draft schema, expert committee)	Australia, 8 states and Commonwealth, purchasers and providers of alcohol and other drug treatment	Ritter et al. (2016)
Procurement regulations and welfare logic	Marketisation, procurement, user involvement	Qualitative content analysis (documents as procurement laws and guidelines, interviews of key persons in 2017)	Denmark, Finland, Norway, and Sweden, market and welfare logic, addiction treatment	Stenius & Storbjörk (2020)
The effects of incentives in a public addiction treatment system	Value-based purchasing, P4P	Qualitative; quasi-experimental pre-post design (admission and discharge data from 2005 to 2011)	Maine, USA, substance use treatment	Stewart et al. (2018)
The effects of the market logic to service provision and service-users	Marketisation, procurement, purchasing	Several data sources of 6 sub-studies of a larger study (statistics from the 1970s, 152 interviews, observation)	Sweden, substance abuse treatment	Storbjörk & Stenius (2019a)
The effects of political ideology on purchasing services	NPM, privatisation, purchasing	Multiple regression models, official statistics from 1970 to 2014	Sweden, addiction treatment system	Storbjörk & Stenius (2019b)
Perceptions of purchasing and payment mechanism	Purchasing, payment for outcome; PbR	Qualitative; semi-structured interviews (197 interviewed in 2013)	Australia, 8 states, and Commonwealth alcohol and other drug treatment system	Van De Ven et al. (2020)
A structural features and vulnerability of alcohol and other drug treatment providers	Procurement arrangements, competitive tendering, NPM	An online survey for managers of treatment providers (47 government and 160 NGO providers in 2018–2019)	Australia, publicly funded AOD treatment providers (specialised)	Van De Ven et al. (2021)

*Note.* AOD = alcohol and other drug; NPM = new public management; P4P = payment by performance; PbR = payment by results.

## Results

### Four models of how drug treatment services are procured

This systematic review identified four general models of how DTS are procured: traditional procurement, value-based procurement, outcome-based procurement, and innovative procurement. Those models can be classified into two categories: the first two are NPM oriented, the third and fourth are NPG oriented. The models differ in how they emphasise design specifications (inputs, process) and performance specifications (outputs, quality, outcomes) (Martin, 2005). Actually, many design specifications, such as personnel, facilities, equipment, and materials, are often guided by country-specific legislation (Pelkonen & Valovirta, 2015; Stenius & Storbjörk, 2020). As a result of differing emphasis, the mechanisms used and other factors of the models differ. In practice, these procurement models are alternative ways to organise publicly funded services such as DTS. Thus they provide solutions for different situations, environments, and goals. Additionally, the models differ in how people can influence the services as citizens or service-users. Despite their differences, the four models also have similarities, and they partly overlap (Table 2).

**Table 2.** Models, mechanisms, and other factors of DTS procurement.

Models, mechanisms, and other factors in the DTS procurement	
Traditional	- Emphasis on design specifications (inputs and process) and performance specifications (outputs, i.e., productised services) - Performance measurement (outputs, e.g., volume of services or customers) - Fee for service instead of a lump sum - DTS users as object or consumer-customers - DTS providers as competitors
Value-based	- Emphasis on performance specifications, especially quality (e.g., waiting time and retention), evidence-based treatment often as a premise - Performance measurement (quality, e.g., waiting time and retention) - P4P, incentives (bonuses and/or penalties) - DTS users as objects of professional's operations - DTS providers as professionals who compete for incentives
Outcome-based	- Emphasis on performance specifications, especially outcomes (client outcomes) - Performance measurements (outcomes, e.g., interventions initiated, waiting time, status at treatment discharge, re-presenting for treatment, abstinence, injecting, housing problems) - PbR, i.e., client outcomes representing recovery - DTS users self-reported certain outcomes, but no other mention of involvement - DTS providers as co-designers who compete for incentives
Innovative	- Emphasis on performance specifications, especially on outcomes (innovations) and on interaction in the first phases of procurement (e.g., seminars and workshops within market dialogue such as space for innovation, a negotiated procedure in tendering) - Performance measurement (outputs, e.g., the volume of services or customers) - Fee for service - DTS users were asked their views in the market dialogue, but no other mention of involvement - DTS providers as a partner to the procurer but a competitor to another provider

*Note.* DTS = drug treatment service; P4P = payment by performance; PbR = payment by results.

In three studies, the description of the procurement corresponded to the traditional procurement model (Stenius & Storbjörk, 2020; Storbjörk & Stenius, 2019a, 2019b). In traditional forms of procurement, the procurer often precisely defines the services procured and the means of procurement: in other words, they are productised (Martin, 2005; Pelkonen & Valovirta, 2015). The model highlights market logic as a steering mechanism and uses competitive tendering in the selection of provider. The contract model is often a framework contract, and the payment model is a fee provided for a service. Within this model, service providers compete with each other. Citizens, as DTS users, are seen as consumer-customers (Stenius & Storbjörk, 2020; Storbjörk & Stenius, 2019a, 2019b). In the research literature, those factors are described as tools the of NPM (Ritter et al., 2016; Storbjörk & Stenius, 2019a, 2019b; Van De Ven et al., 2020, 2022).

The second model is value-based procurement, which we found in two studies conducted in the United States. The model was first implemented in a medical care context but is later also evident in publicly funded substance use treatment. This model emphasis shifts from inputs, process, and outputs towards the definitions of quality of services (see Table 2). A key element of the model is performance measurement, with indicators being defined by the procurer. The other essential elements are financial incentives (bonuses and penalties) and incentive-based contracting (Reif et al., 2021; Stewart et al., 2018). Evidence-based methods seem to be a premise in the services, and the procurer can use purchase levers to support their use (Rieckmann et al., 2011; see also Stenius & Storbjörk, 2020).

The third model identified is the outcome-based model. This model was explored in two studies from the UK, where payment by results (PbR) was piloted as a mechanism of outcome-based commissioning. This model emphasises clearly defined outcomes, and payment was linked to outcome incentives, which were assumed to represent recovery. A co-design group was used to develop the indicators for outcome measurements. The group included representatives from service producers, government departments, and other experts in the field. The studies did not mention whether DTS users were involved in the co-design. However, they were expected to self-report certain outcomes using a validated instrument called a Treatment Outcome Profile (Gosling, 2018; Jones et al., 2018).

The fourth model is called the innovative model. We identified this model in one study where researchers investigated innovative procurement of social care services for substance users in Helsinki, Finland. The procurement sought to create a new service model and new types of services. Implementing the innovative procurement model requires an emphasis on interaction during all phases of the procurement process. In this case, the procurement is implemented using a negotiated procedure. The process starts with market dialogues using seminars and workshops with the city and service providers. The DTS users were also asked for their opinions and expectations of the service and about their needs. Following the market dialogues, the service providers were invited to the negotiation phase that the study reported created space for innovation. The next phase was a call for tenders, at which point the required services were specified in detail. Different award criteria (e.g., innovativeness and quality) were applied to the different service categories (Pelkonen & Valovirta, 2015).

In addition, we found a study that described the conceptual schema for procurement of services to treat alcohol and other forms of drug dependency in Australia. The three dimensions of the schema were as follows: ways in which providers were chosen (e.g., open or targeted competitive tendering); ways in which services were paid for (e.g., block grant, fee for service, or payment for outcome); and ways in which price was managed (fixed or negotiated price). All the mechanisms of the dimensions were adopted in Australia but also in the UK and USA (Ritter et al., 2016). From the perspective of service providers and purchasers, any mix of mechanisms of conceptual schema have strengths but also weaknesses (Van De Ven et al., 2020, 2022). It is notable that DTS user involvement in the procurement processes was not particularly visible, even in the conceptual schema from Australia. The mechanisms of the schema were also included in the four general models identified in this study.

### The goals of drug treatment service procurement and assessments of their attainment

As follows, we focus on the goals of DTS procurement in the four procurement models. In addition, we were interested in how the achievement of goals was assessed. The main results are summarised in Table 3. In the case of studies where the procurement was in line with the traditional model, the goals were described in only a general fashion. Those studies refer in the first place to key factors of NPM that were expected to improve the efficiency, quality, and accountability of publicly funded DTS. Management by results and performance measurements were mentioned as practices of NPM (Stenius & Storbjörk, 2020; Storbjörk & Stenius, 2019a, 2019b). Further, the goals of the procurement were also linked to the country-specific procurement legislation of the Nordic countries and the relevant EU directives. Open, fair, and well-regulated competition was emphasised as a way to deliver the best balance of price and quality of services (Stenius & Storbjörk, 2020).

**Table 3.** The goals of DTS procurement and their assessment.

	Goals and their assessment
Traditional	General goals of NPM – improved cost efficiency, quality, and accountability of publicly funded DTS The goals derived from the procurement legislation (e.g., best relation between price and services) Also, goals derived from social and healthcare legislation and the needs of the citizens
Value-based	Improved quality of services and control of costs Incentivised performance measurement (bonuses and penalties) linked to payment Measurements of waiting time, treatment engagement, retention, etc.
Outcome-based	Improved client outcomes to foster recovery (the domains of national framework: progression towards abstinence, reduction in offending, improved health and wellbeing) PbR by using standardised client outcomes (e.g., the waiting time, status at treatment discharge, re-presenting for treatment, abstinence, injecting, housing problems) DTS users self-reporting tool was also used
Innovative	Creating the new services and innovations and diversity of service portfolio to meet the cross-cutting needs of the service-users Activate and engage the providers and develop partnerships Outcomes can be assessed only by piloting services with service-users

*Note.* DTS = drug treatment service; NPM = new public management.

In the value-based procurement model, the goal is twofold. The first part of the goal is to improve the quality of the services, and the second part involves controlling costs. Compared to traditional procurement, this model does not rely on competitive markets and legislation producing targeted performance to instil better quality. Therefore, in the value-based model, performance-based contracting, performance measurements, and incentivised payment for service producers (bonuses and penalties) are also used. Measurements were used as a tool to establish the goals and assess their performances. Measurements also related to the incentivised payment for the services, which was expected to improve service quality. The indicators for measurements included waiting time for treatment, treatment engagement, and retention as factors of recovery and improved outcomes. The producers also received bonuses when the number of service-users increased (Reif et al., 2021; Stewart et al., 2018).

In outcome-based DTS procurement, the goal is to improve client outcomes to foster recovery. A procurer applying this model does not give very specific instructions on how the services should be produced but tends instead to set out expected results and impacts. In these studies, a national outcomes framework was used that also considered local factors. The three domains of the framework are progression towards abstinence, reduction in offending, and improved health and well-being. Payment is linked to results or, in other words, to standardised client outcomes. Examples of outcome indicators are presented in Table 3. The outcome-based model also issued DTS users a self-reporting tool known as a treatment outcomes profile to help assess outcomes (Gosling, 2018; Jones et al., 2018).

The goal of innovative procurement was first to create new services and service innovations and to enhance the diversity of the service portfolio to address the cross-cutting needs of the service-users. The other goal was to engage service providers in the process and nurture partnerships with the public commissioner. In addition, the purpose was also to reduce costs. The outcomes and impacts of new or innovative services can only be assessed by piloting services with service-users in the later phase of the procurement (Pelkonen & Valovirta, 2015).

The goals of the procurement are often determined as a part of country-specific NPM-oriented health reforms (Van De Ven et al., 2020, 2022). The study authors also state that the systems should be governed with the citizens’ best interests in mind (Storbjörk & Stenius, 2019a, 2019b). Similarly, it was argued that maximising the health and well-being of service-users requires well-informed decisions and an understanding of procurement (Ritter et al., 2016).

### The outcomes of drug treatment service procurement

Finally, we examined the outcomes of the DTS procurement. We were especially interested in the outcomes related to well-being, social inclusion, and harm reduction at the individual level or in society. The main results are summarised in Table 4.

**Table 4.** The outcomes of DTS procurement.

	Outcomes of the DTS procurement
Traditional	A challenge to democracy, equity, and the assessment of general or individual needs New risks linked to accountability and monitoring Undermine cooperation, increases fragmentation May increase marginalisation of service-users or downplay professionals’ values
Value-based	Value-based model, performance contracts, and incentives do not improve quality of treatment Incentives did not reduce waiting time for treatment Incentives have little or no impact on treatment engagement, retention, or completion Financial incentives did not cause the selection of service-users not intended to be targeted
Outcome-based	PbR, as a part of the outcome-based model, does not improve recovery outcomes Waiting time for treatment was longer, treatment completion and completion without re-presenting were lower Abstinence and non-injecting during the treatment programme was higher Changes the working life and roles of treatment professionals
Innovative	Several service innovations, new services, and improvements of existing services were created: more diversified service portfolio and better availability of services, producing the services closer to users’ everyday life and better accessibility to services Competition between providers increased, and the collaboration reduced The service system was still fragmented, and services were not integrated

*Note.* DTS = drug treatment service; PbR = payment by results.

Those studies illustrating procurement resembling the traditional model stated that procurement and purchasing had become the new normal. Procurement did not seem to be ideologically motivated; the reasons for procuring were often quite practical (Storbjörk & Stenius, 2019b). At the system level of outcomes, Storbjörk and Stenius (2019a) describe how competition and procurement undermine cooperation and increase fragmentation. In addition, there are fundamental risks related to the accountability required by legislation. The same authors point out that responsibility for the well-being of the population could not be devolved to private service providers. Finally, purchasing through procurement could also increase the marginalisation of DTS users and downplay professionals’ value (Storbjörk & Stenius, 2019a).

In the value-based model, performance contracts and incentives did not improve the quality of treatment; for example, incentives did not appear to reduce treatment waiting times (Stewart et al., 2018). Incentives had little or no impact on treatment engagement, retention, or completion (Reif et al., 2021). It was apparent that in the addiction treatment system, service user engagement and treatment retention were complex issues and were susceptible to changes in the environment, such as financial hardship and increased use of drugs (Stewart et al., 2018). Under such circumstances, implementing a new procurement or purchasing model requiring systemic changes is extremely challenging (Reif et al., 2021).

The outcome-based model focuses on the payment mechanism, particularly PbR, which is problematic because the outcome-based model also includes other essential elements such as co-production and co-creation with service-users and other stakeholders. The studies analysed do not report that PbR improves recovery outcomes. The waiting time for treatment was longer in those DTS where the payment mechanism was implemented than it was for services where it was not used. In addition, rates of treatment completion and completion without re-presenting were lower than other services. However, abstinence and not injecting during treatment rates were higher than in other services (Jones et al., 2018). Payment by results also seems to impact the working life of treatment professionals and how they perceive their role. It has been argued that economic values have become more important than people's well-being and the needs or the values of professionals or service users (Gosling, 2018). It has also been argued that mechanisms such as PbR, where the time period is relatively short, are not suitable for complex and long-lasting processes such as recovery (Jones et al., 2018).

The innovative procurement form adds several service innovations, new services, and improvements to existing services. One potential system-level innovation was the so-called multi-supplier model, which was created through market dialogue but was not successful in the later phase of the procurement. The most evident benefit of innovative procurement was a more diversified service portfolio. From the perspective of DTS users, this means that there were more services available for different needs. A diverse portfolio could also make it easier to find the appropriate services. The services produced also more closely reflected the everyday lives of DTS users, which might improve the accessibility of the services. Service providers perceived the innovative form of procurement increased competition and reduced collaboration, which caused some providers financial problems so severe that they were forced to cease trading. When the multi-supplier model did not materialise, the DTS systems remained fragmented, and services were not integrated. When the services have been implemented, it will be possible to assess the value for the service-users (Pelkonen & Valovirta, 2015).

In addition to the model-specific outcomes, we found similar emphases in other studies too. First, policy and purchasing levers are incorporated into procurement processes and contracts to support the implementation of methods. However, it is unclear how these methods will ultimately be introduced or what their impact on DTS will be. The reviewed studies point out several factors, including collaboration between stakeholders (including service-users), which could facilitate implementation (Rieckmann et al., 2011). Second, a significant proportion of treatment is delivered by private service providers; however, they are more haphazard than public providers because of competition processes, shorter contracts, and less secure funding mechanisms. Researchers argue that there are fundamental problems with outcome-based funding, which transforms treatment professionals into administrators and service-users into passive recipients. These factors influence the whole service system and its ability to comprehensively meet the needs of service-users (Van De Ven et a., 2020, 2022). Even if the procurement exercises are widespread, there seems to be a lack of research, especially on competitive tendering as a mechanism for choosing providers. Additionally, research on the concepts related to how providers are chosen and paid has often used inadequate methods. However, researchers have argued that it is in the interest of governments to make informed decisions about all aspects of the procurement of these complex services to foster the health and well-being of individuals and society (Ritter et al., 2016).

This study identifies four different DTS procurement models. The results are summarised in Table 5.

**Table 5.** Summary of the results.

Traditional model	Value-based model	Outcome-based model	Innovative model
Goals: Cost efficiency, quality, and accountability of publicly funded DTS	Goals: Quality services and control of the costs	Goals: Client outcomes aiding recovery	Goals: Innovative or new services for the needs of DTS users Engage the providers and develop partnerships
Emphasis on: Performance specifications (outputs) Performance measurement (e.g., volume of productised services) Fee for service DTS users: objects or consumer-customers Providers: competitors	Emphasis on: Performance specifications (quality), evidence-based methods as a premise Performance indicators and measurement P4P and incentives DTS users: objects of professional operations Providers: competitors	Emphasis on: Performance specifications (outcomes) Performance indicators and measurements PbR and incentives DTS users: self-reporting outcomes (e.g., well-being), but no mention of co-creation Providers: competitors, no mention of co-creation excluding the co-design of indicators	Emphasis on: Performance specifications (outcomes) Collaboration through procurement phases Performance measurement (e.g., the volume of services) Fee for service DTS users: their views were asked, but no mention of co-creation Providers: partners to the procurer, competitors to other providers
Outcomes: A challenge to democracy, equity, and assessment of needs of population and individuals Risks linked to accountability and monitoring Undermine cooperation, increases fragmentation May increase DTS users’ marginalisation Downplay professionals’ values	Outcomes: Did not improve quality Incentives did not reduce waiting time for treatment and had little impact or none at all on treatment engagement, retention, or completion Incentives did not cause unintended selection (cherry-picking) of service-users	Outcomes: Did not improve recovery outcomes Waiting time for treatment was longer, treatment completion and completion without re-presenting were lower Abstinence and non-injecting in the treatment was higher Changes the working life and roles of professionals	Outcomes: Service innovations and new services were created Competition between providers increased, and the collaboration reduced The DTS system was still or even more fragmented, and services were not integrated Value for DTS users can only be evaluated after piloting the innovative or new services

*Note.* DTS = drug treatment service; P4P = payment for performance; PbR = payment by results.

## Conclusions and a further research agenda

The main motivation of this systematic review was to unearth how DTS are procured and the goals and outcomes of such procurement operations. The review was conducted using the PRISMA approach (Moher et al., 2009) and the PICO framework. The analysis used content analysis (e.g., Mikkonen & Kääriäinen, 2019) and the expanded systems framework (Martin, 2005).

This study shows that there is no single form of DTS procurement used in OECD countries but many kinds. However, although different forms of DTS procurement are common and affect individuals and wider communities, we found only 12 empirical studies (1990–2020) focusing on them. In addition, despite the excellent conceptual scheme presented by Ritter et al. (2016), many studies define the concept of public procurement and its related concepts poorly or do not define them at all. Further, the focus of studies is often limited to payment mechanisms. Consequently, although DTS are procured for people in need of care and support, knowledge of their perspectives and their involvement as co-creators in the procurement phases was not advanced by many of the studies reviewed.

The key findings of this study shed light on the goals, emphasis, and outcomes of four overlapping DTS procurement models. This knowledge is needed to organise the inherently complex DTS in a way that creates value for both service-users and society. The reviewed studies suggest that the mechanisms of the traditional model simplified the complex DTS to present them as a product that can compete in the market. The model increased fragmentation and caused challenges for the equity and accountability required by legislation, and it may also increase the marginalisation of DTS users (Stenius & Storbjörk, 2020; Storbjörk & Stenius, 2019a; see also Van De Ven et al., 2020, 2022). These findings align with previous literature that describes the fundamental challenges around an NPM orientation in a complex environment (e.g., Osborne, 2021, Raisio et al., 2019). The value-based model seeks to improve the quality of DTS with performance indicators linked to financial incentives. However, these quite linear and professional-oriented mechanisms did not improve the quality of complex DTS (Reif et al., 2021; Stewart et al., 2018).

The outcome-based model seeks to improve DTS outcomes to foster recovery. In the literature, co-production and co-creation of value are essential to the outcome-based ideology (e.g., Osborne, 2021; Tirronen, 2020), but surprisingly the reviewed studies do not confirm that theorised importance. Instead, they focus on performance indicators linked to payment that were also used in the outcome-based model. And again, these mechanisms did not improve the outcomes to foster recovery. The innovative model aims to create services to meet the multiple needs of DTS users. Meeting that aim requires co-creation with DTS users and other stakeholders in all procurement phases. The articles reviewed report the early phases of procurement feature seminars, workshops, and negotiated procedures. The DTS users were asked for their views, but there is no other mention of their involvement in co-creation. The collaboration between providers also reduced, and competition increased, during the later phases of this procurement model. Although individual service innovations emerged, the DTS system remained fragmented (Pelkonen & Valovirta, 2015).

**Figure 4.**
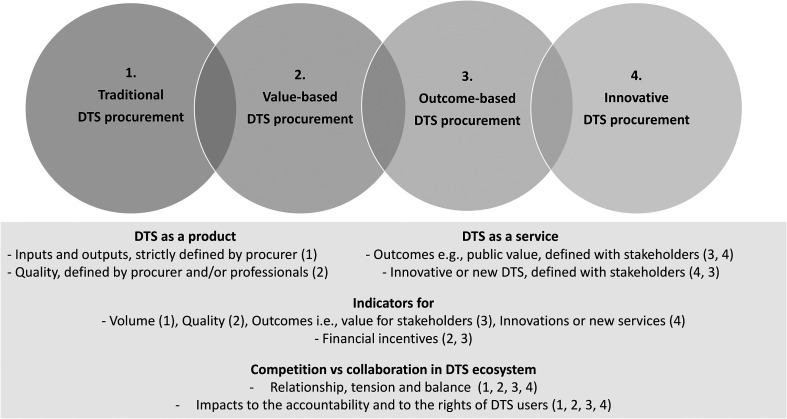
The pointers to guide the drug treatment service procurement.

One important lesson from this study is that neither competition nor financial incentives improve the efficiency, quality, or outcomes of complex DTS. Another is that although co-production and co-creation of value are essential factors of the outcome-based and innovative models, there is a lack of knowledge on their implementation in the phases of complex DTS procurement. These pointers should guide managers, policy-makers, practitioners, and researchers to pay attention to the following questions (see Figure 4): Is the DTS system understood and managed like a complex issue, and is the difference between product and service logic clear? Is the emphasis on inputs and outputs or value co-creation with DTS users and other stakeholders? Are indicators used to measure the experiences of, and the value for, DTS users? How might the tension between competition and collaboration in the complex DTS system be addressed? And lastly, what are the potential impacts of different procurement models on the accountability that is required by legislation?

The findings of this study do have their limitations. To assess the limitations of the empirical studies reviewed in this study, we used a checklist for qualitative and quantitative studies, and the criteria used were mainly fulfilled. The information on ethical issues was, however, often not applicable. One limitation of our study is that some potential studies were excluded owing to not being peer-reviewed and written in English or Finnish. The grey literature was excluded because of the challenge of searching for it systematically. To minimise the limitations linked to research terms, we conducted test searches and consulted specialists.

There can be little doubt that more research on DTS procurement is needed, particularly on co-creation of the value with and for DTS users and other stakeholders. The above findings of this systematic review are significant to consider in future research. International collaboration between researchers to combine and further develop the conceptual schema (Ritter et al., 2016) and the DTS procurement models described in this study could also be fruitful.

## Supplemental Material

sj-docx-1-nad-10.1177_14550725231157503 - Supplemental material for Drug treatment service procurement: A systematic review of models, goals, and outcomes Click here for additional data file. Supplemental material, sj-docx-1-nad-10.1177_14550725231157503 for Drug treatment service procurement: A systematic review of models, goals, and outcomes by Taina Schneider, Kristiina Kuussaari and Petri Virtanen in Nordic Studies on Alcohol and Drugs

sj-docx-2-nad-10.1177_14550725231157503 - Supplemental material for Drug treatment service procurement: A systematic review of models, goals, and outcomes Click here for additional data file. Supplemental material, sj-docx-2-nad-10.1177_14550725231157503 for Drug treatment service procurement: A systematic review of models, goals, and outcomes by Taina Schneider, Kristiina Kuussaari and Petri Virtanen in Nordic Studies on Alcohol and Drugs
