# Supplementary material for: Drug treatment service procurement: A systematic review of models, goals, and outcomes
Source: Nordisk Alkohol Nark. 2023 Jun 22;40(5):424–42. doi: 10.1177/14550725231157503 (PMC10634391; doi:10.1177/14550725231157503)
Supplement: sj-docx-2-nad-10.1177_14550725231157503 - Supplemental material for Drug treatment service procurement: A systematic review of models, goals, and outcomes [file sj-docx-2-nad-10.1177_14550725231157503.docx]

**Appendix 2**

**Quality assesment**

The quality checklist was developed based on existing guidelines and checklists for qualitative and quantitative studies (Spencer et.al., 2003; Tranfield et al., 2003; Rojon et al., 2021; Tufanaru et al., 2020).

1=Fulfilled excellently, 2=Fulfilled well, 3=Fulfilled moderately, 4=Fulfilled poorly ja 5=Not applicable

| Study | Purpose and aim | Theory | Research question | Design/ congruence | Sample | Data collection | Analysis | Findings | Control group | Multiple measurements | Measured the same way | Measured in a reliable way | Appropriate statistical analysis | Ethics |
| --- | --- | --- | --- | --- | --- | --- | --- | --- | --- | --- | --- | --- | --- | --- |
| **1.** | 1 | 1,5 | 3 | 1,5 | 1 | 1 | 1 | 1 | 5 | 5 | 5 | 5 | 5 | 5 |
| **2.** | 1 | 2 | 3 | 1,5 | 1 | 1 | 1 | 1 | 1 | 1 | 1 | 1 | 1 | 3,5 |
| **3.** | 1 | 1 | 1 | 1,5 | 3 | 1,5 | 1,5 | 1 | 5 | 5 | 5 | 5 | 5 | 3,5 |
| **4.** | 1 | 1,5 | 1 | 1 | 1 | 1 | 1 | 1 | 5 | 5 | 5 | 2 | 1 | 2,5 |
| **5.** | 1 | 1,5 | 2 | 1,5 | 2 | 2 | 1,5 | 1 | 5 | 5 | 5 | 5 | 5 | 1 |
| **6.** | 1 | 1 | 1 | 1 | 1,5 | 1,5 | 1,5 | 1,5 | 5 | 5 | 5 | 5 | 5 | 5 |
| **7.** | 1 | 1,5 | 1 | 1 | 1 | 1 | 1 | 1 | 1 | 1 | 1 | 1 | 1 | 1 |
| **8.** | 1 | 1 | 1 | 1,5 | 1,5 | 1,5 | 1,5 | 1,5 | 5 | 5 | 5 | 5 | 5 | 4 |
| **9.** | 1 | 1 | 1 | 1 | 1,5 | 1,5 | 1,5 | 2 | 5 | 5 | 5 | 5 | 1,5 | 5 |
| **10.** | 1 | 1 | 1 | 1 | 1 | 1 | 1 | 1 | 5 | 5 | 5 | 5 | 5 | 2 |
| **11.** | 1 | 1 | 1 | 1 | 1 | 1 | 1 | 1 | 5 | 5 | 5 | 1 | 1 | 2 |
| **12.** | 1 | 1 | 3 | 1 | 1 | 1 | 1 | 1 | 1,5 | 1 | 1 | 1 | 1 | 3,5 |
